# Supplementary material for: Heat adaptation of phage T7 under an extended genetic code
Source: Virus Evol. 2021 Dec 1;7(2):veab100. doi: 10.1093/ve/veab100 (PMC8923235; doi:10.1093/ve/veab100)
Supplement: veab100_Supp [file veab100_supp.zip › Sequencing_Analysis.zip!/Sequencing_Analysis/S2/evidence/JC_SIDE_2_68.html]

BRESEQ :: Evidence


| New junction evidence | | | | | | | | | | | |
| --- | --- | --- | --- | --- | --- | --- | --- | --- | --- | --- | --- |
|  | | seq id | position | reads (cov) | reads (cov) | score | skew | freq | annotation | gene | product |
| \* | ? | NC\_001604.1 | = 16604 | 3197 (0.600) | 1690 (0.320) | 335/590 | 1.7 | 34.5% | intergenic (–/–) | *–/–* | –/– |
|  | ? | NC\_001604.1 | 17028 = | 3275 (0.630) |  |  |  |  | intergenic (–/–) | *–/–* | –/– |

Only 100 of 5164 total aligned reads displayed.  

`TGACATTCTTGCGGTTGTGGTTGACGAAGATGTGCTTATTTTCACATGGGACGAAGTAAAAGGTAAGACATCCATGTGTGTCGGCAAGAGAAACAAAGGCATAAAACTATAGGAGAAATTATTATGGCTATGACAAAGAAATTTAAAGTGTCCTTCGACGTTACCGCAAAGATGTCGTCTGACGTTCAGGCAATCTTAGAGAAAGATATGCTGCATCTATGTAAGCAGGTCGGCTCAGGTGCGATTGTCCCCAATGGTAAACAGAAGGAAATGATTGTCCAGTTCCTGACACACGGTATGGAAGGATTGATGACATTCGTAGTACGTACATCATTTCGTGAGGCCATTAAGGACATGCACGAAGAGTATGCAGATAAGGACTCTTTCAAACAATCTCCTGCAACAGTACGGGAGGTGTTCTGATGTCTGACTACCTGAAAGTGCTGCAAGCAATCAAAAGTTGCCCTAAG‑ACTTTCCAGTCCAACTATGTACGGAACAATGCGAGCCTCGTAGCGGAGGCCGCTTCCCGTGGTCACATCTCGTGCCTGACTACTAGTGGACGTAACGGTGGCGCTTGGGAAATCACTGCTTCCGGTACTCG  >  NC_001604.1/16728‑17328`  

                                                                                                                                                                                                                                                                                                            `|`                                                                                                                                                                                                                                                                                                               
     `ttCTTGCGGTTGTGGTTGACGAAGATGTGCTTATTTTCACATGGGACGAAGTAAAAGGTAAGACATCCATGTGTGTCGGCAAGAGAAACAAAGGCATAAAACTATAGGAGAAATTATTATGGCTATGACAAAGAAATTTAAAGTGTCCTTCGACGTTACCGCAAAGATGTCGTCTGACGTTCAGGCAATCTTAGAGAAAGATATGCTGCATCTATGTAAGCAGGTCGGCTCAGGTGCGATTGTCCCCAATGGTAAACAGAAGGAACTGATTGTCCAGTTCCTGACACACGGTATGGAAGGa                                                                                                                                                                                                                                                                                                          >  1:673157/1‑301 (MQ=255)`  
     `ttCTTGCGGTTGTGGTTGACGAAGATGTGCTTATTTTCACATGGGACGAAGTAAAAGGTAAGACATCCATGTGTGTCGGCAAGAGAAACAAAGGCATAAAACTATAGGAGAAATTATTATGGCTATGACAAAGAAATTTAAAGTGTCCTTCGACGTTACCGCAAAGATGTCGTCTGACGTTCAGGCAATCTAAGAGAAAGATATGCTGCATCTATGTAAGCAGGTCGGCTCAGGTGCGATTGTCCCCAATGTTAAACAGAAGGAAATGATTGTCCAGTTCCGGACACACGGTATGGAAGGa                                                                                                                                                                                                                                                                                                          >  1:455282/1‑301 (MQ=255)`  
             `atccaagtaggctgccgtaccgaagagattgctcaggtggtcattgagaccgcacaagaagcgatgcgctgggttggagaccactggaacttccggtgtcttctggataccgaaggtaagatgggtcctaattgggcgatttgccactgatacaggaggctactcatgaacgaaagacacttaacaggtgctgcttctgaaatgctagtagcctacaaatttaccaaagctgggtacactgtctattaccctatgctgactcagagtaaagaggacttggttgtatgGAAGGATTGATGAc                                                                                                                                                                                                                                                                                                  <  1:609639‑M2/14‑1 (MQ=255)`  
               `tgtgGTTGACGAAGATGTGCTTATTTTCACATGGGACGAAGTAAAAGGTAAGACATCCATGTGTGTCGGCAAGAGAAACAAAGGCATAAAACTATAGGAGAAATTATTATGGCTATGACAAAGAAATTTAAAGTGTCCTTCGACGTTACCGCAAAGATGTCGTCTGACGTTCAGGCAATCTTAGAGAAAGATATGCTGCATCTATGTAAGCAGATCGGCTCAGGTGCGATTGTCCCCAATGGTAAAAAGAAGGAAATGATTGTCCAGTTCCTGACACAAGTTATGGAAGGATTGATGACAt                                                                                                                                                                                                                                                                                                >  1:282959/1‑301 (MQ=255)`  
                        `cGAAGATGTGCTTATTTTCACATGGGACGAAGTAAAAGGTAAGACATCCATGTGTGTCGGCAAGAGAAACAAAGGCATAAAACTATAGGAGAAATTATTATGGCTATGACAAAGAAATTTAAAGTGTCCTTCGACGTTACCGCAAAGATGTCGTCTGACGTTCAGGCAATCTTAGAGAAAGATATGCTGCATCTATGTAAGCAGGTCGGCTCAGGTGCGATTGTCCCCAATGGTAAACAGAAGGAAATGATTGTCCAGTTCCTGACACAAGGTATGGAAGGATTGATGAGATTCgtagctg                                                                                                                                                                                                                                                                                       >  1:765143/1‑298 (MQ=255)`  
                          `aaGATGTGCTTATTTTCACATGGGACGAAGTAAAAGGTAAGACATCCATGTGTGTCGGCAAGAGAAACAAAGGCATAAAACTATAGGAGAAATTATTATGGCTATGACAAAGAAATTTAAAGTGTCCTTCGACGTTACCGCAAAGATGTCGTCTGACGTTCAGGCAATCTTAGAGAAAGATATGCTGCATCTATGTAAGCAGGTCGGCTCAGGTGCGATTGTCCCCAATGGTAAACAGAAGGAAATGATTGTCCAGTTCCTGACACACGGTATGGAAgctgtctcttatacacatctccga                                                                                                                                                                                                                                                                                     >  1:688163/1‑278 (MQ=255)`  
                               `gtgCTTATTTTCACATGGGACGAAGTAAAAGGTAAGACATCCATGTGTGTCGGCAAGAGAAACAAAGGCATAAAACTATATGAGAAATTATTATGGCTATGACAAAGAAATTTAAAGTGTCCTTCGACGTTACCGCAAAGATGTCGTCTGACGTTCAGGCAATCTTAGAGAAAGATATGCTGCATCTATGTAAGCAGGTCGGCTCAGGTGCGATTGTCCCCAATGGTAAACAGAAGGAAATGATTGTCCAGTTCCTGACACACGGTATGGAAGGATTGATGACATTCGTAGTACGTAcatc                                                                                                                                                                                                                                                                                <  1:790528/301‑1 (MQ=255)`  
                                    `tATTTTCACATGGGACGAAGTAAAAGGTAAGACATCCATGTGTGTCGGCAAGAGAAACAAAGGCATAAAACTATAGGAGAAATTATTATGGCTATGACAAAGAAATTTAAAGTGTCCTTCGACGTTACCGCAAAGATATCGTCTGACGTTCAGGCAATCTTAGAGAAAGATATGCTGCATCTATGTAAGCAGGTCGGCTCAGGTGCGATTGTCCCCAATGGTAAACAGAAGGAAATGATTGTCCAGTTCCTGACACACGGTATGGAAGGATTGATGACATTCGTAGTACGTACATCATTTc                                                                                                                                                                                                                                                                           >  1:336301/1‑301 (MQ=255)`  
                                      `ttttCACATGGGACGAAGTAAAAGGTAAGACATCCATGTGTGTCGGCAAGAGAAACAAAGGCATAAAACTATAGGAGAAATTATTATGGCTATGACAAAGAAATTTAAAGTGTCCTTCGACGTTACCGCAAAGATGTCGTCTGACGTTCAGGCAATCTTAGAGAAAGATATGCTGCATCTATGTAAGCAGGTCGGCTCAGGTGCGATTGTCCCCAATGGTAAACAGAAGGAAATGATTGTCCAGTTCCTGACACACGGTATGGAAGGATTGATGACATTCGTAGTACGTACATCATTTCGt                                                                                                                                                                                                                                                                         >  1:441021/1‑301 (MQ=255)`  
                                       `ggagatgtgtataagagacagaGGTAAGACATCCATGTGTGTCGGCAAGAGAAACAAAGGCATAAAACTATAGGAGAAATTATTATGGCTATGACAAAGAAATTTAAAGTGTCCTTCGACGTTACCGCAAAGATGTCGTCTGACGTTCAGGCAATCTTAGAGAAAGATATGCTGCATCTATGTAAGCAGGTCGGCTCAGGTGCGATTGTCCCCAATGGTAAACAGAAGGAAATGATTGTCCAGTTCCTGACACACGGTATGGAAGGATTGATGACATTCGTAGTACGTACATCATTTCGTg                                                                                                                                                                                                                                                                        <  1:644162/280‑1 (MQ=255)`  
                                                 `aaCGAAGTAAAAGGTAAGACATCCATGTGTGTCGGCAAGAGAAACAAAGGCATAAAACTATAGGAGAAATTATTATGGCTATGACAAAGAAATTTAAAGTGTCCTTCGACGTTACCGCAAAGATGTCGTCTGACGTTCAGGCAATCTTAGAGAAAGATATGCTGCATCTATGTAAGCAGGTCGGCTCAGGTGCGATTGTCCCCAATGGTAAACAGAAGGAAATGATTGTCCAGTTCCTGACACACGGTATGGAAGGATTGATGACATTCGTAGTACGTACATCATTTCGTGAGGCCATTaa                                                                                                                                                                                                                                                              <  1:298408/300‑1 (MQ=255)`  
                                                  `aCGAAGTAAAAGGTAAGACATCCATGTGTGTCGGCAAGAGAAACAAAGGCATAAAACTATAGGAGAAATTATTATGGCTATGACAAAGAAATTTAAAGTGTCCTTCGACGTTACCGCAAAGATGTCGTCTGACGTTCAGGCAATCTTAGAGAAAGATATGCTGCATCTATGTAAGCAGGTCGGCTCAGGTGCGATTGTCCCCAATGGTAAACAGAAGGAAATGATTGTCCAGTTCCTGACACACGGTATGGAAGGATTGATGACATTCGTAGTACGTACATCATTTCGTGAGGCCATTAAg                                                                                                                                                                                                                                                             <  1:756977/301‑1 (MQ=255)`  
                                                  `aCGAAGTAAAAGGTAAGACAGCCATGTGTGTCGGAAAGAGAAACAAAGGCATAAAACTATAGGAGAAATTATTATGGCTATGACAAAGAAATTTAAAGTGTCCTTCGACGTTACCGCAAAGATGTCGTCTGACGTTCAGGCAATCTTAGAGAAAGATATGCTGCATCTATGTAAGCAGGTCGGCTCAGGTGCGATTGTCCCCAATGGTAAACAGAAGGAAATGATTGTCCAGTTCCTGACACACGGTATGGAAGGATTGATGACATTCGTAGTACGTACATCATTTCGTGAGGCCATTAAg                                                                                                                                                                                                                                                             <  1:805411/301‑1 (MQ=255)`  
                                                   `gatgtgtataagagacagaTCCATGTGTGTCGGCAAGAGAAACAAAGGCATAAAACTATAGGAGAAATGATTATGGCTATGACAAAGAAATTTAAAGTGTCCTTCGACGTTACCGCAAAGATGTCGTCTGACGTTCAGGCAATCTTAGAGAAAGATATGCTGCATCTATGTAAGCAGGTCGGCTCAGGTGCGATTGTCCCCAATGGTAAACAGAAGGAAATGATTGTCCAGTTCCTGACACACGGTATGGAAGGATTGATGACATTCGTAGTACGTACATCATTTCGTGAGGCCATTAAgg                                                                                                                                                                                                                                                            <  1:496281/283‑1 (MQ=255)`  
                                                    `gtcattgagaccgcacaagaagcgatgcgctgggttggagaccactggaacttccggtgtcttctggataccgaaggtaagatgggtcctaattgggcgatttgccactgatacaggaggctactcatgaacgaaagacacttaacaggtgctgcttctgaaatgctagtagcctacaaatttaccaaagctgggtacactgtctattaccctatgctgactcagagtaaagaggacttggttgtatgGAAGGATTGATGACATTCGTAGTACGTACATCATTTCGTGAGGCCATTAAGGa                                                                                                                                                                                                                                                           >  1:170046‑M2/249‑301 (MQ=255)`  
                                                     `aaGTAAAAGGTAAGACATCCATGTGTGTCGTCAAGAGAAACCAAGGCATAAAACTATAGGAGAAATTATTATGTCTATGACAAAGACATTTAAAGTGTCCTTCGACGTTACCGCAAAAATGTCGTCTGGCGTTCAGGCAATCTTAGAGAAAGATATTCTGCATCTATGTAAGCAGGTCGGCTCATGTTCGATTGTCCCCAATGGTAAACCGAAGGCAATGATTATCCAGTTCCTGACACACGGTATGGAAGGATTGCTGACATTCATAGTACGTACATCATTTCGTTAGCCCATTAAGGAc                                                                                                                                                                                                                                                          >  1:109073/1‑301 (MQ=255)`  
                                                      `aGTAAAAGGTAAGACATCCATGTGTGTCGGCAAGAGAAACAAAGGCATAAAACTATAGGAGAAATTATTATGGCTATGACAAAGAAATTTAAAGTGTCCTTCGACGTTACCGCAAAGATGTCGTCTGACGTTCAGGCAATCTTAGAGAAAGATATGCTGCATCTATGTAAGCAGGTCGGCTCAGGTGCGATTGTCCCCAATGGTAAACAGAAGGAAATGATTGTCCAGTTCCTGACACACGGTATGGAAGGATTGATGACATTCGTAGTACGTACATCATTTCGTGAGGCCATTAAGGACa                                                                                                                                                                                                                                                         <  1:275225/301‑1 (MQ=255)`  
                                                       `gTAAAAGGTAAGACATCCATGTGTGTCGGCAAGAGAAACAAAGGCATAAAACTATAGGAGAAATTATTATGGCTATGACAAAGAAATTTAAAGTGTCCTTCGACGTTACCGCAAAGATGTCGTCTGACGTTCAGGCAATCTTAGAGAAAGATATGCTGCATCTATGTAAGCAGGTCGGCTCAGGTGCGATTGTCCCCAATGGTAAACAGAAGGAAATGATTGTCCAGTTCCTGACACACGGTATGGAAGGATTGATGACATTCGTAGTCCGTACATCATTTctgtctcttatacacatatc                                                                                                                                                                                                                                                        >  1:266987/1‑282 (MQ=255)`  
                                                       `gTAAAAGGTAAGACATCCATGTGTGTCGGCAAGAGAAACAAAGGCATAAAACTATAGGAGAAATTATTATGGCTATGACAAAGAAATTTAAAGTGTCCTTCGACGTTACCGCAAAGATGTCGTCTGACGTTCAGGCAATCTTAGAGAAAGATATGCTGCATCTATGTAAGCAGGTCGGCTCAGGTGCGATTGTCCCCAATGGTAAACAGAAGGAAATGATTGTCCAGTTCCTGACACACGGTATGGAAGGATTGATGACATTCGTAGTACGTACATCATTTCGTGAGGCCATTAAGGACAt                                                                                                                                                                                                                                                        >  1:390131/1‑301 (MQ=255)`  
                                                          `aagggTAAGACATCCATGTGTGTCGGCAAGAGAAACAAAGGCATAAAACTATAGGAGAAATTATTATGGCTATGACAAAGAAATTTAAAGTGTCCTTCGACGTTACCGCAAAGATGTCGTCTGACGTTCAGGCAATCTTAGAGAAAGATATGCTGCATCTATGTAAGCAGGTCGGCTCAGGTGCGATTGTCCCCAATGGTAAACAGAAGGAAATGATTGTCCAGTTCCTGACACACGGTATGGAAGGATTGATGACATTCGTAGTACGTACATCATTTCGTGAGGCCATTAAGGACATGCa                                                                                                                                                                                                                                                     <  1:47990/298‑1 (MQ=255)`  
                                                            `aGGTAAGACATCCATGTGTGTCGGCAAGAGAAACAAAGGCATAAAACTATAGGAGAAATTATTATGGCTATGACAAAGAAATTTAAAGTGTCCTTCGACGTTACCGCAAAGATGTCGTCTGACGTTCAGGCAATCTTAGAGAAAGATATGCTGCATCTATGTAAGCGGGTCGGCTCAGGTGCGATTGTCCCCAATGGTAAACAGAAGGAAATGATTGTCCAGTTCCTGACACACGGTATGGAAGGATTGATGACATTCGTAGTACGTACATCATTTCGTGAGGCCATTAAGGACATGCACg                                                                                                                                                                                                                                                   >  1:420305/1‑301 (MQ=255)`  
                                                             `ggTAAGACATCCATGTGTGTCGGCAAGAGAAACAAAGGCATAAAACTATAGGAGAAATTATTATGGCTATGACAAAGAAATTTAAAGTGTCCTTCGACGTTACCGCAAAGATGTCGTCTGACGTTCAGGCAATCTTAGAGAAAGATATGCTGCATCTATGTAAGCAGGTCGGCTCAGGTGCGATTGTCCCCAATGGTAAACAGAAGGAAATGATTGTCCAGTTCCTGACACACGGTATGGAAGGATTGATGACATTCGTAGTACGTACATCATTTCGTGAGGCCATTAAGGACATGCACGa                                                                                                                                                                                                                                                  >  1:305618/1‑301 (MQ=255)`  
                                                             `agTAAGACATCCATGTGTGTCGGCAAGAGAAACAAAGGCATAAAACTATAGGAGAAATTATTATGGCTATGACAAAGAAATTTAAAGTGTCCTTCGACGTTACCGCAAAGATGTCGTCTGACGTTCAGGCAATCTTAGAGAAAGATATGCTGCATCTATGTAAGAAGGTCGGCTCAGGTGCGATTGTCCCCAATGGTAAACAGAAGGAAATGATTGTCCAGTTCCTGACACACGGTATGGAAGGATTGATGACATTCGTAGTACGTACATCATTTCGTGAGGCCATTAAGGACATGCACGa                                                                                                                                                                                                                                                  <  1:381291/300‑1 (MQ=255)`  
                                                               `cgcacaagaagcgatgcgctgggttggagaccactggaacttccggtgtcttctggataccgaaggtaagatgggtcctaattgggcgatttgccactgatacaggaggctactcatgaacgaaagacacttaacaggtgctgcttctgaaatgctagtagcctacaaatttaccaaagctgggtacactgtctattaccctatgctgactcagagtaaagaggacttgattgtatgGAAGGATTGATGACATTCGTAGTACGTACATCATTTCGTGAGGCCATTAAGGACATGCACGAag                                                                                                                                                                                                                                                <  1:26044‑M2/64‑1 (MQ=255)`  
                                                                 `aGACATCCATGTGTGTCGTCAAGAGAAACAAAGGCATAAAACTATAGGAGAAATTATTATGGCTATGACAAAGAAATTTAAAGTGTCCTTCGACGTTACCGCAAAGATGTCGTCTGACGTTCAGGCAATCTTAGAGAAAGATATGCTGCATCTATGTAAGCAGGTCGGCTCAGGTGCGATTGTCCCCAATGGTAAACAGAAGGAAATGATTGTCCAGTTCCTGACACACGGTATGGAAGGATTGATGACATTCGTAGTACGTACATCATTTCGTGAGGCCATTAAGGACATGCACGAagag                                                                                                                                                                                                                                              <  1:289589/301‑1 (MQ=255)`  
                                                                 `aGACATCCATGTGTGTCGGCAAGAGAAACAAAGGCATAAAACTATAGGAGAAATTATTATGGCTATGACAAAAAAATTTAAAGTGTCCTTCGACGTTACCGCAAAGATGTCGTCTGACGTTCAGGCAATCTTAGAGAAAGATATGCTGCATCTATGTAAGCAGGTCGGCTCAGGTGCGATTGTCCCCAATGGTAAACAGAAGGAAATGATTGTCCAGTTCCTGACACACGGTATGGAAGGATTGATGACATTCGTAGTACGTACATCATTTCGTGAGGCCATTAAGGACATGCACGAagag                                                                                                                                                                                                                                              <  1:161973/301‑1 (MQ=255)`  
                                                                   `aCATCCATGTGTGTCGGCAAGAGAAACAAAGGCATAAAACTATAGGAGAAATTATTATGGCTATGACAAAGAAATTTAAAGTGTCCTTCGACGTTACCGCAAAGATGTCGTCTGACGTTCAGGCAATCTTAGAGAAAGATATGCTGCATCTATGTAAGCAGGTCGGCTCAGGTGCGATTGTCCCCAATGGTAAACAGAAGGAAATGATTGTCCAGTTCCTGACACCCGGTATGGAAGGATTGATGACATTCGTAGTACGTAAATCATTTCGTGAGGCCATTAAGGACATGCACGAAGAGTa                                                                                                                                                                                                                                            >  1:216307/1‑301 (MQ=255)`  
                                                                     `aTCCATGTGTGTCGGCAAGAGAAACAAAGGCATAAAACTATAGGAGACATTATTATGGCTATGACAAAGAAATTTAAAGTGTCCTTCGACGTTACCGCAAAGATGTCGTCTGACGTTCAGGCAATCTTAGAGAAAGATATGCTGCATCTATGTAAGCAGGTCGGCTCAGGTGCGATTGTCCCCAATGGTAAACAGAAGGAAATGATTGTCCAGTTCCTGACACACGGTATGGAAGGATTGATGACATTCGTAGTACGTACATCATTTCGTGAGGCCATTAAGGACATGCACGAAGAGTAtt                                                                                                                                                                                                                                          >  1:750825/1‑300 (MQ=255)`  
                                                                      `gaagcgatgagctgggtgtgataccaaaggaacttccggtgtgttctggataccgaaggtaagatgggtcataattgggcgatttgacactgatacaggaggctactcatgaacgaaagacaattaacaggtgatgcttctgaaatgctagtagcctacaaatttaccaaagctgggtacactgtctattaccctatgatgactcagagtaaagaggacgtggttgtatgGAAGGATTGATGACATTCGTAGTACGTACATCATTTCGTGAGGCCATTAAGGACATGCACGAAGAGTATGc                                                                                                                                                                                                                                         <  1:811564‑M2/71‑1 (MQ=255)`  
                                                                        `cATGTGTGTCGGCAAGAGAAACAAAGGCATAAAACTATAGGAGAAATTATTATGGCTATGACAAAGAAATTTAAAGTGTCCTTCGACGTTACCGCAAAGATGTCGTCTGACGTTCAGGCAATCTTAGAGAAAGATATGCTGCATCTATGTAAGCAGGTCGGCTCAGGTGCGATTGTCCCCAATGGTAAACAGAAGGAAATGATTGTCCAGTTCCTGACACACGGTATGGAAGGATTGATGACATTCGTAGTACGTACATCATTTCGTGAGGCCATTAAGGACATGCACGAAGAGTATGCAg                                                                                                                                                                                                                                       <  1:624187/301‑1 (MQ=255)`  
                                                                         `gcgatgcgctgggttggagaccactggaacttccggtgtcttctggataccgaaggtaagatgggtcctaattgggcgatttgccactgatacaggaggctactcatgaacgaaagacacttaacaggtgctgcttctgaaatgctagtagcctacaaatttaccaaagctgggtacactgtctattaccctatgctgactcagagtaaagaggacttggttgtatgGAAGGATTGATGACATTCGTAGTACGTACATCATTTCGTGAGGCCATTAAGGACATGCAAGAAGAGTATGCAGa                                                                                                                                                                                                                                      <  1:586277‑M2/74‑1 (MQ=255)`  
                                                                           `gtgtgtCGGCAAGAGAAACAAAGGCATAAAACTATAGGAGAAATTATTATGGCTATGACAAAGAAATTTAAAGTGTCCTTCGACGTTACCGCAAAGATGTCGTCTGACGTTCAGGCAATCTTAGAGAAAGATATGCTGCATCTATGTAAGCAGGTCGGCTCAGGTACGATTGTCCCCAATGGTAAACAGAAGGAAATGATTGTCCAGTTCCTGACACACGGTATGGAAGGATTGATGACATTCGTAGTACGTACATCATTTCGTGAGGCCATTAAGGACATGCACGAagagctgtctctta                                                                                                                                                                                                                                    >  1:549585/1‑291 (MQ=255)`  
                                                                                `taggCAAGAGAAAAAAAGGCAGAAAACTATAGGAGAAATTATTATGGCTATGACAAAGAAATTTAAAGTGTCCTTCGACGTTACCGCAAAGATGTCGTCTGACGTTCAGGCAATCTTAGAGAAAGATATGCTGCATCTATGTAAGCAGGTCGGCTCAGGTGCGATTGTCCCCAATGGTAAACAGAAGGAAATGATTGTCCAGTTCCTGACACACGGTATGGAAGGATTGATGACATTCGTAGTACGTACATCATTTCGTGAGGCCATTAAGGACATGCACGAAGAGTATGCAGATAAGGAc                                                                                                                                                                                                                               <  1:771072/299‑1 (MQ=255)`  
                                                                                `tCGGCAAGAGAAACAAAGGCATAAAACTATAGGAGAAATTATTATGGCTATGACAAAGAAATTTAAAGTGTCCTTCGACGTTACCGCAAAGATGTCGTCTGACGTTCAGGCAATCTTAGAGAAAGATATGCTGCATCTAGGTAAGCAGGTCGGCTCAGGTGCGATTGTCCCCAATGGTAAACAGAAGGAAATGATTGTCCAGTTCCTGACACACGGGATGGAAGGATTGATGACATTCGTAGTACGTACATCATTTCGTGAGGCCATTAAGGACATGCACGAAGAGTATGCAGATAAGGAc                                                                                                                                                                                                                               <  1:329115/301‑1 (MQ=255)`  
                                                                                            `acacAGGCATAAAACTATAGGAGAAATTATTATGGCTATGACAAAGAAATTTAAAGTGTCCTTCGACGTTACCGCAAAGATGTCGTCTGACGTTCAGGCAATCTTAGAGAAAGATATGCTGCATCTATGTAAGCAGGTCGGCTCAGGTGCGATTGTCCCCAATGGTAAACAGAAGGAAATGATTGTCCAGTTCCTGACACACGGTATGGAAGGATTGATGAAATTCGTAGTACGTACATCATTTCGTGCGGCCATTAAGGACATGCACGAAGCGTATGCAGATAAGGAcctgtctcttata                                                                                                                                                                                                                   >  1:557224/1‑289 (MQ=255)`  
                                                                                              `aaaGGCATAAAACTATAGGAGAAATTATTATGGCTATGACAAAGAAATTTAAAGTGTCCTTCGACGTTACCGCAAAGATGTCGTCTGACGTTCAGGCAATCTTAGAGAAAGATATGCTGCATCTATGTAAGCAGGTCGGCTCAGGTGCGATTGTCCCCAATGGGAAACAGAAGGAAATGATTGTCCAGTTCCTGACACACGGTATGGAAGGATTGATGACATTCGTAGTACGTACATCATTTCGTGAGGCCATTAAGGACATGCACGAAGAGTATGCAGATAAGGACTCTTTCAAACAAtc                                                                                                                                                                                                                 <  1:343056/301‑1 (MQ=255)`  
                                                                                                  `gCATAAAACTATAGGAGAAATTATTATGGCTATGACAAAGAAATTTAAAGTGTCCTTCGACGTTACCGCAAAGATGTCGTCTGACGTTCAGGCAATCTTAGAGAAAGATATGCTGCATCTATGTAAGCAGGTCGGCTCAGGTGCGATTGTCCCCAATGGTAAACAGAAGGAAATGATTGTCCAGTTCCTGACACACGGTATGGAAGGATTGATGACATTCGTAGTACGTACATCATTTCGTGAGGCCATTAAGGACATGCACGAAGAGTATGCAGATAAGGACTCTTTCAAACAATCTCCt                                                                                                                                                                                                             >  1:636466/1‑301 (MQ=255)`  
                                                                                                      `cttctggtgtcttctggataccgaaggtaagatgggtcctaattgggcgatttgccactgatacaggaggctactcatgaacgaaagacacttaacaggtgctgcttctgaaatgctagtagcctacaaatttaccaaagctgggtacactgtctattaccctatgctgactcagagtaaagaggacttggttgtatgGAAGGATTGATGACATTCGTAGTACGTACATCATTTCGTGAGGCCATTAAGGACATGCACGAAGAGTATGCAGATAAGGACTATTTCAAACAATCTCCTGCaa                                                                                                                                                                                                         >  1:722044‑M2/199‑301 (MQ=255)`  
                                                                                                      `cttccggtgtcttctggataccgaaggtaagatgggtcctaattgggcgatttgccactgatacaggaggctactcatgaacgaaagacacttaacaggtgctgcttctgaaatgctagtagcctacaaatttaccaaagctgggtacactgtctattaccctatgctgactcagagtaaagaggacttggttgtatgGAAGGATTGATGACATTCGTAGTACGTACATCATTTCGTGAGGCCATTAAGGACATGCACGAAGAGTATGCAGATAAGGACTCTTTCAAACAATATCCTGCaa                                                                                                                                                                                                         >  1:71394‑M2/199‑301 (MQ=255)`  
                                                                                                      `aaaaCTATAGGAGAAATTATTATGGCTATGACAAAGAAATTTAAAGTGTCCTTCGACGTTACCGAAAAGATGTCGTCTGACGTTCAGGCAATCTTAGAGAAAGATAGGCTGCATCTATGGAAGCAGGTCGGCTCAGGTGCGATTGTCCCCAATGGTAAACAGAAGGAAATGATTGTCCAGTTCCTGACACACGGTATGGAAGGATTGATGACATTCGTAGTACGTACATCATTTCGTGAGGCCATTAAGGACATGCACGAAGAGTATGCAGATAAGGACTCTTTCAAACAATCTCCTGCaa                                                                                                                                                                                                         <  1:797590/301‑1 (MQ=255)`  
                                                                                                       `aaaCTATAGGAGAAATTATTATGGCTATGACAAAGAAATTTAAAGTGTCCTTCGACGTTACCGCAAAGATGTCGTCTGACGTTCAGGCAATCTTAGAGAAAGATATGCTGCATCTATGTAAGCAGGTCGGCTCAGGTGCGATTGTCCCCAATGGTAAACAGAAGGAAATGATTGTCCAGATCCTGACACACGGTATGGAAGGATTGATGACATTCGTAGTACGTACATCATTTCGTGAGGCCATTAAGGACATGCACGAAGAGTATGCAGATAAGGACTCTTTCAAAcctgtctcttatac                                                                                                                                                                                                        >  1:729819/1‑288 (MQ=255)`  
                                                                                                          `cTATAGGAGAAATTATTATGGCTATGACAAAGAAATTTAAAGTGTCCTTCGACGTTACCGCAAAGATGTCGTCTGACGTTCAGGCAATCTTAGAGAAAGATATGCTGCATCTATGTAAGCAGGTCGGCTCAGGTGCGATTGTCCCCAATGGTAAACAGAAGGAAATGATTGTCCAGTTCCTGACACACGGTATGGAAGGATTGATGACATTCGTAGTACGTACATCATTTCGTGAGGCCATTAAGGACATGCACGAAGAGTATGCAGATAAGGACTCTTTCAAACAATCTCCTGCAACAGt                                                                                                                                                                                                     >  1:519848/1‑301 (MQ=255)`  
                                                                                                                 `agaAATTATTATGGCTATGACAAAGAAATTTAAAGTGTCCTTCGACGTTACCGCAAAGATGTCGTCTGACGTTCAGGCAATCTTAGAGAAAGATATGCTGCATCTATGTAAGCAGGTCGGCTCAGGTGCGATTGTCCCCAATGGTAAACAGAAGGAAATGATTGTCCAGTTCCTGACACACGGTATGGAAGTATTGATGACATTCGTAGTACGTACATCATTTCGTGAGGCCATTAAGGACATGCACGAAGAGTATGCAGATAAGGACTCTTTCAAACAATCTCCTGCAACAGTACGGGAg                                                                                                                                                                                              <  1:117295/301‑1 (MQ=255)`  
                                                                                                                  `gggagatgtgtataagagacagggtcctaattgggcgatttgccactgatacaggaggctactcatgaacgaaagacacttaacaggtgctgcttctgaaatgctagtagcctacaaatttaccaaagctgggtacactgtctattaccctatgctgactcagagtaaagaggacttggttgtatgGAAGGATTGATGACATTCGTAGTACGTACATCATTTCGTGAGGCCATTAAGGACATGCACGAAGAGTATGCAGATAAGGACTCTTTCAAACAATCTCCTGCAACAGTACGGGAgg                                                                                                                                                                                             <  1:358664‑M2/115‑1 (MQ=255)`  
                                                                                                                  `aaaaTTATTAGGGCTATGACAAAGAAATTGAAAGTGTCCTTCGACGTTACCGCAAAGATGTCGTCTGACGTTCAGGCAATCTTAGAGAAAGATATGCTGCATCTATGTAAGCAGGTCGTCTCAGGTGCGATTGTCCCCAATGGTAAACAGAAGGAAATGATTGTCCAGTTCCTGACACACGGTATGGAAGGATTGATGACATTCGTAGTACGTACATCATTTCGTGAGGCCATTAAGGACATGCACGAAGAGTATGCAGATAAGGACTCTTTCAAACAATCTCCTGCAACAGTACGGGAgg                                                                                                                                                                                             <  1:23815/300‑1 (MQ=255)`  
                                                                                                                   `aaaTTATTATGGCTATGACAAAGAAATTTAAAGTGTCCTTCGACGTTACCGCAAAGATGTCGTCTGACGTTCAGGCAATCTTAGAGAAAGATATGCTGCATCTATGTAAGCAGGTCGGCTCAGGTGCGATTGTCCCCAATGGTAAACAGAAGGAAATGATTGTCCAGTTCCTGACACACGGTATGGAAGGATTGATGACATTCGTAGTACGTACATCATTTCGTGAGGCCATTAAGGACATGCACGAAGAGTATGCAGATAAGGACTCTTTCAAACAATCTCCTGCAACAGTACGGGAGgt                                                                                                                                                                                            <  1:145887/301‑1 (MQ=255)`  
                                                                                                                          `tgtataagagacagggtcctaattgggcgatttgccactgatacaggaggctactcatgaacgaaagacacttaacaggtgctgcttctgaaatgctagtagcctacaaatttaccaaagctgggtacactgtctattaccctatgctgactcagagtaaagaggacttggttgtatgGAAGGATTGATGACATTCGTAGTACGTACATCATTTCGTGAGGCCATTAAGGACATGCACGAAGAGTATGCAGATAAGGACTCTTTCAAACAATCTCCTGCAACAGTACAGGAGGTGTTCTGa                                                                                                                                                                                     <  1:783463‑M2/123‑1 (MQ=255)`  
                                                                                                                          `tatGGCTATGACAAAGAAAGTTAAAGTGGTCTTCGACGTTACCGCAAAGATGTCGTCTGACGTTCAGGCAATCTTAGAGAAAGATATGCTGCATCTATGTAAGCAGGTCGGCTCAGGTGCGATTGTCCCCAATGGTAAACAGAAGGAAATGATTGTCCAGTTCCTGACACACGGTATGGAAGGATTGATGACATTCGTAGTACGTACATCATTTCGTGAGGCCATTAAGGACATGCACGAAGAGTATGCAGATAAGGACTCTTTCAAACAATCTCCTGCAACAGTACGGGAGGTGTTCTGa                                                                                                                                                                                     <  1:826435/301‑1 (MQ=255)`  
                                                                                                                          `acagaggtaagatgggtcctaattgggcgatttgccactgatacaggaggctactcatgaacgaaagacacttaacaggtgctgcttctgaaatgctagtagcctacaaatttaccaaagatgggtacactgtctattaccctatgctgactcagagtaaagaggacttggttgtatgGAAGGATTGATGACATTCGTAGTACGTACATCATTTCGTGAGGCCAGTAAGGACATGCACGAAGAGTATGCAGATAAGGACTCTTTCAAACAATCTCCTGCAACAGTACGGGAGGTGTTCTGa                                                                                                                                                                                     <  1:427079‑M2/123‑1 (MQ=255)`  
                                                                                                                             `ggCTATGACAAAGAAATTTAAAGTGTCCTTCGACGTTACCGCAAAGATGTCGTCTGACGTTCAGGCAATCTTAGAGAAAGATATGCTGCATCTATGTAAGCAGGTCGGCTCAGGTGCGATTGTCCCCAATGGTAAACAGAAGGAAATGATTGTCCAGTTCCTGACACACGGTATGGAAGGATTGATGACATTCGTAGTACGTACATCATTTCGTGAGGCCATTAAGGACATGCACGAAGAGTATGCAGATAAGGACTCTTTCAAACAATCTCCTGCAAcctgtctcttatacacatctccg                                                                                                                                                                                  >  1:564166/1‑279 (MQ=255)`  
                                                                                                                               `ggtaagatgggtcctaatttggcgatttgccactgatacaggaggctactcatgaacgaaagacacttaacaggtgctgcttctgaaatgctagtagcctacaaatttaccaaagctgggtacactgtctattaccctatgctgactcagagtaaagaggacttggttgtatgGAAGGATTGATGACATTCGTAGTACGTACATCATTTCGTGAGGCCATTAAGGACATGCACGAAGAGTATGCAGATAAGGACTCTTTCAAACAATCTCCTGcaacagtacgggaggctgtctcttatac                                                                                                                                                                                >  1:25316‑M2/174‑288 (MQ=255)`  
                                                                                                                                           `aaTTTAAAGTGTCCTTCGACGTTACCGCAAAGATGTCGTCTGACGTTCAGGCAATCTTAGAGAAAGATATGCTGCATCTATGTAAGCAGGTCGGCTCAGGTGCGATTGTCCCCAATGGTAAACAGAAGGAAATGATTGTCCAGTTCCTGACACACGGTATGGAAGGATTGATGACATTCGTAGTACGTACATCATTTCGTGAGGCCATTAAGGACATGCACGAAGAGTATGCAGATAAGGACTCTTTCAAACAATCTCCTGCAACAGTACGGGAGGTGTTCTGATGTCTGACTACCTGaca                                                                                                                                                                    >  1:52766/1‑299 (MQ=255)`  
                                                                                                                                            `gggagatgtgtataagagacagaCCGCAAAGATGTCGTCTGACGTTCAGGCAATCTTAGAGAAAGATATGCTGCATCTATGTAAGCAGGTCGGCTCAGGTGCGATTGTCCCCAATGGTAAACAGAAGGAAATGATTGTCCAGTTCCTGACACACGGTATGGAAGGATTGATGACATTCGTAGTACGTACATCATTTCGTGAGGCCATTAAGGACATGCACGAAGAGTATGCAGATAAGGACTCTTTCAAACAATCTCCTGCAACAGTACGGGAGGTGTTCTGATGTCTGACTACCTGAAAg                                                                                                                                                                   <  1:593758/279‑1 (MQ=255)`  
                                                                                                                                             `taatttggcgatttgccactgatacaggaggctactcatgaacgaaagacacttaacaggtgctgcttctgaaatgctagtagcctacaaatttaccaaagctgggtacactgtctattaccctatgctgactcagagtaaagaggacttggttgtatgGAAGGATTGATGACATTCGTAGTACGTACATCATTTCGTGAGGCCATTAAGGACATGCACGAAGAGTATGCAGATAAGGACTCTTTCAAACAATCTCCTGCAACAGTACGGGAGGTGTTCTGATGTCTGACTACCTGAAAGt                                                                                                                                                                  >  1:650769‑M2/160‑301 (MQ=255)`  
                                                                                                                                                      `tCCTTCGACGTTACCGCAAAGATGTCGTCTGACGTTCAGGCAATCTTAGAGAAAGATATGCTGCATCTATGTAAGCAGGTCGGCTCAGGTGCGATTGTCCCCAATGGTAAACAGAAGGAAATGATTGTCCAGTTCCTGACACACGGTATGGAAGGATTGATGACATTCGTAGTACGTACATCATTTCGTGAGGCCATTAAGTACATGCACGAAGAGTATGCAGATAAGGACTCTTTCAAACAATCTCCTGCAACAGTACGGGAGGTGTTCTGATGTCTGACTACCTGAAAGTGCTgcaagc                                                                                                                                                         <  1:503572/301‑1 (MQ=255)`  
                                                                                                                                                                `ttACCGCAAAGAGGTCGTCTGACGGTCAGGCAATCTTAGAGAAAGATATGCTGCATCTATGTAAGCAGGTCGGCTCAGGTGCGATTGTCCCCAATGGTAAACAGAAGGAAATGATTGTACAGTTCCTGACACACGGTATGGAAGGATTGATGACATTCGTAGTACGTACATCATTTCGTGAGGCCATTAAGGACATGCACGAAGAGTATGCAGATAAGGACTCTTTCAAACAATCTCCTGCAACAGTACGGGAGGTGTTCTGATGTCTGACTACCTGAAAGTGCTGCAAGCAATCAAAAGt                                                                                                                                               <  1:85844/301‑1 (MQ=255)`  
                                                                                                                                                                `tgatacaggaggctactcatgaacgaaagacacttaacaggtgctgcttctgaaatgctagtagcctacaaatttaccaaagctgggtacactgtctattaccctatgctgactcagagtaaagaggacttggttgtatgGAAGGATTGATGACATTCGTAGTACGTACATCATTTCGTGAGGCCATTAAGGACATGCACGAAGAGTATGCAGATAAGGACTCTTTCAAACAATCTCCTGCAACAGTACGGGAGGTGTTCTGATGTCTGACTACCTGAAAGTGCTGCAAGCAATCAAAagg                                                                                                                                               >  1:579040‑M2/141‑300 (MQ=255)`  
                                                                                                                                                                `tgatacaggaggctactcatgaacgaaagacacttaacaggtgctgcttctgaaatgctagtagcctacaaatttaccaaagctgggtacactgtctattaccctatgctgactcagagtaaagaggacttggttgtatgGAAGGATTGATGACATTCGTAGTACGTACATCATTTCGTGAGGCCATTAAGGACATGCACGAAGAGTATGCAGATAAGGACTCTTTCAAACAATCTCCTGCAACAGTACGGGAGGTGTTCTGATGTCTGACTACCTGAAAGTGCTGCAAGCAATCAAAAGt                                                                                                                                               <  1:696352‑M2/161‑1 (MQ=255)`  
                                                                                                                                                                      `cAAAGATGTCGTCTGACGTTCAGGCAATCTTAGAGAAAGATATGCTGCATCTATGTAAGCAGGTCGGCTCAGGTGCGATTGTCCCCAATGGTAAACAGAAGGAAATGATTGTCCAGTTCCTGACACACGGTATGGAAGGATTGATGACATTCGTAGTACGTACATCATTTCGTGAGGCCATTAAGGACATGCACGAAGAGTATGCAGATAAGGACTCTTTCAAATAATCTCCTGCAACAGTACGGGGGGTGTTCTGATGTTTGACTACCTGAAAGTCCTGCAAGCCATCAAAATTTGCCCt                                                                                                                                         >  1:320594/1‑301 (MQ=255)`  
                                                                                                                                                                           `gagacagcTGACGTTCAGGCAATCTTAGAGAAAGATAGGCTGCATCTATGTAAGCAGGTTGGCTCAGGTGCGATTGTCCCCAATGGTAAACAGAAGGAAATGATTGTCCAGTTCCTGACACACGGTATGGAAGGATTGATGACATTCGTAGTACGTACATCATTTCGTGAGGCCATTAAGGACATGCACGAAGAGAATGCAGATAAGGACTCTTTCAAACAATCTCCTGCAACAGTACGGGAGGTGTTCTGATGTCTGACTACCTGAAAGTGCTGCAAGCAATCAAAAGTTGCCCTAAG‑Ac                                                                                                                                   <  1:245218/294‑1 (MQ=255)`  
                                                                                                                                                                           `aTGGCGTCTGACGTGCAGGCAATCTTAGAGAAAGATATGCTGCAGCTATGTAAGCAGGTCGGCTCAGGTGCGATTGTCCCCAATGGTAAACAGAAGGAAATGATTGTCCAGTTCCTGACACACGGTATGGAAGGATTGATGACATTCGTAGTACGTACATCATTTCGTGAGGCCATTAAGGACATGCACGAAGAGTATGCAGATAAGGACTCTTTCAAACAATCTCCTGCAACAGTACGGGAGGTGTTCTGATGTCTGACTACCTGAAAGTGCTGCAAGCAATCAAAAGTTGCCCTAAG‑Ac                                                                                                                                   <  1:600864/301‑1 (MQ=255)`  
                                                                                                                                                                                         `aaagacacttaacaggtgctgcttctgaaatgctagtagcctacaaatttaccaaagctgggtacactgtctattaccctatgctgactcagagtaaagaggacttggttgtatgGAAGGATTGATGACATTCGTAGTACGTACATCATTTCGTGAGGACATTAAGGACATGCACGAAGAGTATGCAGATAAGGACTCTTTCAAACAATCTCCTGCAACAGTACGGGAGGTGTTCTGATGTCTGACTACCTGAAAGTGCTGCAAGCAATCAAAAGTTGCCCTAAG‑ACTTTCCAGTCCAACt                                                                                                                     >  1:70605‑M2/116‑301 (MQ=255)`  
                                                                                                                                                                                          `cAGGCAATCTTAGAGAAAGATATGCTGCATCGATGTAAGCAGGTCGGCTCAGGTGCGATTGTCACCAATGGTAAACAGAAGGAAATGATTGTCCAGTTCCTGACACACGGTATGGAAGGATTGATGACATTCGTAGTACGTACATCATTTCGTGAGGCCATTAAGGACATGCACGAAGAGTATGCAGATAAGGACTCTTTCAAACAATCTCCTGCAACAGTACGGGAGGTGTTCTGATGTCTGACTACCTGAAAGTGCTGCAAGCAATCAAAAGTTGCCCTAAG‑ACTTTCCAGTCCAACTa                                                                                                                    <  1:616213/301‑1 (MQ=255)`  
                                                                                                                                                                                            `ggCAATCTTAGAGAAAGATATGCTGCATCTATGTAAGCAGGTCGGCTCAGGTGCGATTGTCCCCAATGGTAAACAGAAGGAAATGATTGTCCAGTTCCTGACACACGGTATGGAAGGATTGATGACATTCGTAGTACGTACATCATTTCGTGAGGCCATTAAGGACATGCACGAAGAGTATGCAGATAAGGACTCTTTCAAACAATCTCCTGCAACAGTACGGGAGGTGTTCTGATGTCTGACTACCTGAAAGTGCTGCAAGCAATCAAAAGTTGCCCTAAG‑ACTTTCCAGTCCAACTATg                                                                                                                  <  1:208431/301‑1 (MQ=255)`  
                                                                                                                                                                                                `aTCTTAGAGAAAGATATGCTGCATCTATGTAAGCAGGTCGGCTCAGGTTCGATTGTCCCCAATGGTAAACAGAAGGAAATGATTGTCCAGTTCCTGACACACGGTATGGAAGGATTGATGACATTCGTCGTACGTACATCATTTCGTGAGGCCATTAAGGACATGCACGAAGAGTATGCAGATAAGGCCTCTTTCAAACAATCTCCTGCAACAGTACGGGAGGTGTTCTGATGTCTGACTACCTGAAAGTGCTGCAAGCAATCAAAAGTTGCCCTAAG‑ACTTTCCAGTCCAACTATGTACg                                                                                                              >  1:351515/1‑301 (MQ=255)`  
                                                                                                                                                                                                `aTCTTAGAGAAAGATATGCTGCATCTATGTAAGCAGGTCGGATCAGGTGCGATTGTCCCCAATGGTAAACAGAAGGAAATGATTGTCCAGTTCCTGACACACGGTATGGAAGGATTGATGACATTCGTAGTACGTACATCATTTCGTGAGGCCATTAAGGACATGCACGAAGAGTATGCAGATAAGGACTCTTTCAAACAATCTCCTGCAACAGTACGGGAGGTGTTCTGATGTCTGACTACCTGAAAGTGCTGCAAGCAATCAAAAGTTGCCCTAAG‑ACTTTCCAGTCCAACTATTTAcc                                                                                                              >  1:192334/1‑300 (MQ=255)`  
                                                                                                                                                                                                    `tAGAGAAAGATATGCTGCATCTATGTAAGCAGGTCGGCTCAGGTGCGATTGTCCCCAATGGTAAACAGAAGGAAATGATTGTCCAGTTCCTGACACACGGTATGGAAGGATTGATGACATTCGTAGTACGTACATCATTTCGTGAGGCCATTAAGGACATGCACGAAGAGTATGCAGATAAGGACTCTTTCAAACAATCTCCTGCAACAGTACGGGAGGTGTTATGATGTCTGACTACCTGAAAGTGATGCAAGCAATCAAAAGTTGCCCTAAG‑ACTTTCCAGTCCAACTATGTACGGAAc                                                                                                          <  1:572787/301‑1 (MQ=255)`  
                                                                                                                                                                                                         `aaaGATATGCTGCATCTATGTAAGCAGGTCGGCTCAGGTGCGATTGTCCCCAATGGTAAACAGAAGGAAATGATTGTCCAGTTCCTGACACACGGTATGGAAGGATTGATGACATTCGTAGTACGTACATCATTTCGTGAGGCCATTAAGGACATGCACGAAGAGTATGCAGATAAGGACTCTTTCAAACAATCTCCTGCAACAGTACGGGAGGTGTTCTGATGTCTGACTACCTGAAAGTGCTGCAAGCAATCAAAAGTTGCCCTAAG‑ACTTTCCAGTCCAACTATGTACGGAACAATGc                                                                                                     <  1:411234/301‑1 (MQ=255)`  
                                                                                                                                                                                                         `aaaGATATGCTGCATCTATATAAGCAGGTCGGCTCAGGTGCGATTGTCCCCAATGGTAAACAGAAGGAAATGATTGTCCAGTTCCTGACACACGGTATGGAAGGATTGATGACATTCGTAGTACGTACATCATTTCGTGAGGCCATTAAGGACATGCACGAAGAGTATGCAGATAAGGACTCTTTCAAACAATCTCCTGCAACAGTACGGGAGGTGTTCTGATGTCTGACTACCTGAAAGTGCTGCAAGCAATCAAAAGTTGCCCTAAG‑ACTTTCCAGTCCAACTATGTACGGAACAATGc                                                                                                     <  1:535010/301‑1 (MQ=255)`  
                                                                                                                                                                                                             `gcttctgaaatgctagtagcctacaaatttaccaaagctgggtacactgtctattaccctatgctgactcagagtaaagaggacttggttgtatgGAAGGATTGATGACATTCGTAGTACGTACATCATTTCGTGAGGCCATTAAGGACATGCACGAAGAGTATGCAGATAAGGACTCTTTCAAACAATCTCCTGCAACAGTACGGGAGGTGTTCTGATGTCTGACTACCTGAAAGTGCTGCAAGCAATCAAAAGTTGCCCTAAG‑ACTTTCCagtccaactatgtactgtctcttatacac                                                                                                 >  1:365877‑M2/96‑287 (MQ=255)`  
                                                                                                                                                                                                             `gcttctgaaatgctagtagcctacaaatttaccaaagctgggtacactgtctattaccctatgctgactcagagtaaagaggacttggttgtatgGAAGGATTGATGACATTCGTAGTACGTACATCATTTCGTGAGGCCATTAAGGACATGCACGAAGAGTATGCAGATAAGGACTCTTTCAAACAATCTCCTGCAACAGTACGGGAGGTGTTCTGATGTCTGACTACCTGAAAGTGCTGCAAGCAATCAAAAGTTGCCCTAAG‑ACTTTCCAGTCCAACTATGTACGGAACAATGCGAGc                                                                                                 >  1:819484‑M2/96‑301 (MQ=255)`  
                                                                                                                                                                                                                      `aTCTATGTAAGCAGGTCGGCTCAGGTGCGATTGTCCCCAATGGTAAACAGAAGGAAATGATTGTCCAGTTCCTGACACACGGTATGGAAGGATTGATGACATTCGTAGTACGTCCATCATTTCGTGAGGCCATTAAGGACATGCACGATGAGTATGCAGATAAGGACTCTTTCAAACAATCTCCTGCAACAGTACGGGATGTGTTCTGATGTCTGACTACCTGAAAGTGCTGCAAGCCATCAAACGTTGCCCTAAG‑CCTTTCCAGTCCAACTATTTACGGCACAATGCGAGCCTCGTcgcg                                                                                        >  1:742719/1‑301 (MQ=255)`  
                                                                                                                                                                                                                         `tATGTAAGCAGGTCGGCTCAGGTGCGATTGTCCCCAATGGTAAACAGAAGGAAATGATTGTCCAGTTCCTGACACACGGTATGGAAGGATTGATGACATTCGTAGTACGTACATCATTTCGTGAGGCCATTAAGGACATGCACGAAGAGTATGCAGATAAGGACTCTTTCAAACAATCTCCTGCAACAGTACGGGAGGTGTTCTGATGTCTGACTACCTGAAAGTGCTGCAAGCAATCAAAAGTTGCCCTAAG‑ACTTTCCAGTCCAACTATGTACGGAACAATGCGATCCTCGTAgaggag                                                                                     >  1:184259/1‑301 (MQ=255)`  
                                                                                                                                                                                                                         `gctcggagatgtgtataagagacagctgggtacactgtctattaccctatgctgactcagagtaaagaggacttggttgtatgGAAGGATTGATGACATTCGTAGTACGTACATCATTTCGTGAGGCCATTAAGGACATGCACGAAGAGTATGCAGATAAGGACTCTTTCAAACAATCTCCTGCAACAGTACGGGAGGTGTTCTGATGTCTGACTACCTGAAAGTGCTGCAAGCAATCAAAAGTTGCCCTAAG‑ACTTTCCAGTCCAACTATGTACGGAACAATGCGAGCCTCGTAGCGGAg                                                                                     <  1:131185‑M2/218‑1 (MQ=255)`  
                                                                                                                                                                                                                                      `cGGCTCAGGTGCGATTGTCCCCAATGGTAAACAGAAGGAAATGATTGTCCAGTTCCTGACACACGGTATGGAAGGATTGATGACATTCGTAGTACGTACATCATTTCGTGAGGCCATTAAGGACATGCACGAAGAGTATGCAGATAAGGACTCTTTCAAACAATCTCCTGCAACAGTACGGGAGGTGTTCTGATGTCTGACTACCTGAAAGTGCTGCAAGCAATCAAAAGTTGCCCTAAG‑ACTTTCCAGTCCAACTATGTACGGAACAATGCGAGCCTCGTAGCGGAGGCCGCTTCCCGTg                                                                        <  1:681818/301‑1 (MQ=255)`  
                                                                                                                                                                                                                                           `cAGGTGCGATTGTCCCCAATGGTAAACAGAAGGAAATGATTGTCCAGTTCCTGACACACGGTATGGAAGGATTGATGACATTCGTAGTACGTACATCATTTCGTGAGGCCATTAAGGACATGCACGAAGAGTATGCAGATAAGGACTCTTTCAAACAATCTCCTGCAACAGTACGGGAGGTGTTCTGATGTCTGACTACCTGAAAGTGCTGCAAGCAATCAAAAGTTGCCCTAAG‑ACTTTCCAGTCCAACTATGTACGGAACAATGCGAGCCTCGTAGCGGAGGCCGCTTCCCGTGGTcac                                                                   >  1:312047/1‑301 (MQ=255)`  
                                                                                                                                                                                                                                              `gTGCGATTGTCCCCAATGGTAAACAGAAGGAAATGATTGTCCAGTTCCTGACACACGGTATGGAAGGATTGATGACATTCGTATTACGTACATCATTTCGTGAGGCCATTAAGGACATGCACGAAGAGTATGCAGATAAGGACTCTTTCAAACAATCTCCTGCAACAGTACGGGAGGTGTTCTGATGTCTGACTACCTGAAAGTGCTGCAAGCAATCAAAAGTTGCCCTAAG‑ACTTTCCAGTCCAACTATGTACGGAACAATGCGAGCCTCGTAGCGGAGGCCGCTTCCCGTGGTCACAtc                                                                <  1:658375/301‑1 (MQ=255)`  
                                                                                                                                                                                                                                               `aagctgggtacactgtctattaccctatgctgactcagagtaaagaggacttggttgtatgGAAGGATTGATGACATTCGTAGTACGTACATCATTTCGTGAGGCCATTAAGGACATGCACGAAGAGTATGCAGATAAGGACTCTTTCAAACAATCTCCTGCAACAGTACGGGAGGTGTTCTGATGTCTGACTACCTGAAAGTGCTGCAAGCAATCAAAAGTTGCCCTAAG‑ACTTTCCAGTCCAACTATGTACGGAACAATGCGAGCCTCGTAGCGGAGGCCGCTTCCCGTGGTCACAtct                                                               <  1:510589‑M2/240‑1 (MQ=255)`  
                                                                                                                                                                                                                                                   `tcggagatgtgtataagagacagggAAATGATTGTCCAGTTCCTGACACACGGTATGGAAGGATTGATGACATTCGTAGTACGTACATCATTTCGTGAGGCCATTAAGGACATGCACGAAGAGTATGCAGATAAGGACTCTTTCAAACAATCTCCTGCAACAGTACGGGAGGTGTTCTGATGTCTGACTACCTGAAAGTGCTGCAAGCAATCAAAAGTTGCCCTAAG‑ACTTTCCAGTCCAACTATGTACGGAACAATGCGAGCCTCGTAGCGGAGGCCGCTTCCCGTGGTCACATCTCGTg                                                           <  1:777509/278‑1 (MQ=255)`  
                                                                                                                                                                                                                                                     `tGTCCCCAATGGTAAACAGAAGGAAATGATTGTCCAGTTCCTGACACACGGTATGGAAGGATTGATGACATTCGTAGTACGTACATCATTTCGTGAGGCCATTAAGGACATGCACGAAGAGTATGCAGATAAGGACTCTTTCAAACAATCTCCTGCAACAGTACGGGAGGTGTTCTGATGTCTGACTACCTGAAAGTGCTGCAAGCAATCAAAAGTTGCCCTAAG‑ACTTTCCAGTCCAACTATGTACGGAACAATGCGAGCCTCGTAGCGGAGGCCGCTTCCCGTGGTCACATCTCGTGcc                                                         <  1:447210/301‑1 (MQ=255)`  
                                                                                                                                                                                                                                                     `cagacactgtctattaccctatgctgactcagagtaaagaggacttggttgtatgGAAGGATTGATGACATTCGTAGTACGTACATCATTTCGTGAGGCCATTAAGGACATGCACGAAGAGTATGCAGATAAGGACTCTTTCAAACAATCTCCTGCAACAGTACGGGAGGTGTTCTGATGTCTGACTACCTGAAAGTGCTGCAAGCAATCAAAAGTTGCCCTAAG‑ACTTTCCAGTCCAACTATGTACGGAACAATGCGAGCCTCGTAGCGGAGGCCGCTTCCCGTGGTCACATCTCGTGcc                                                         <  1:123821‑M2/246‑1 (MQ=255)`  
                                                                                                                                                                                                                                                           `cAATGGTAAACAGAAGGAAATGATTGTCCAGTTCCTGACACACGGTATGGAAGGATTGATGACATTCGTAGTACGTACATCATTTCGTGAGGCCATTAAGGACATGCACGAAGAGTATGCAGATAAGGACTCTTTCAAACAATCTCCTGCAACAGTACGGGAGGTGTTCTGATGTCTGACTACCTGAAAGTGCTGCAAGAAATCAAAAGTTGCCCTAAG‑ACTTTCCAGTCCAACTATTTACGGAACAATGCGATCCTCTTAGCGGAGGCCGCTTCCCTTGGTCCCATCTCGTGCCTGactt                                                   >  1:433983/1‑300 (MQ=255)`  
                                                                                                                                                                                                                                                             `gtctattaccctatgctgactcagagtaaagaggacttggttgtatgGAAGGATTGATGACATTCGTAGTACGTACATCATTTCGTGAGGCCATTAAGGACATGCACGAAGAGTATGCAGATAAGGACTCTTTCAAACAATCTCCTGCAACAGTACGGGAGGTGTTCTGATGTCTGACTACCTGAAAGTGCTGCAAGCAATCAAAAGTTGCCCTAAG‑ACTTTACAGTCCAACTATGTACGGAACAATGCGAGCCTCGTAGCGGAGGCCGCTTCCCGTGGTCACATCTCGTGCCTGactact                                                 >  1:100076‑M2/48‑301 (MQ=255)`  
                                                                                                                                                                                                                                                             `gtctattaccctatgctgactcagagtaaaaaggacttggttgtatgGAAGGATTGATGACATTCGTAGTACGTACATCATTTCGTGAGGCCATTAAGGACATGCACGAAGAGTATGCAGATAAGGACTCTTTCAAACAATCTCCTGCAACAGTACGGGAGGTGTTCTGATGTCTGACTACCTGAAAGTGCTGCAAGCAATCAAAAGTTGCCCTAAG‑ACTTTCCAGTCCAACTATGTACGGAACAATGCGAGCCTCGTAGCGGAGGCCGCTTCCCGTGGTCACACCTCGTGCCTGactact                                                 >  1:666217‑M2/48‑301 (MQ=255)`  
                                                                                                                                                                                                                                                               `ctattaccctatgctgactcagagtaaagaggacttggttgtatgGAAGGATTGATGACATTCGTAGTACGTACATCATTTCGTGAGGCCATTAAGGACATGCACGAAGAGTATGCAGATAAGGACTCTTTCAAACAATCTCCTGCAACAGTAATGGAGGTGTTCTGATGTCTGACTACCTGAAAGTGCTGGAAGAAATCAAAAGTTGCCCTAAG‑ACTTTCCAGTCAAACTATGTACGGAACAATGCGAGCCTAGTAGCGGAGGCCGCTTCCCGTGGTCACAAACCGGTCCTGACGACTAg                                               >  1:488341‑M2/46‑301 (MQ=255)`  
                                                                                                                                                                                                                                                                    `accctatgctgactcagagtaaagaggacttggttgtatgGAAGGATTGATGACATTCGTAGTACGTACATCATTTCGTGAGGCCATTAAGGACATGCACGAAGAGTATGCAGATAAGGACTCTTTCAAACAATCTCCTGCAACAGTACGGGAGGTGTTCTGATGTCTGACTACCTGAAAGTGCTGCAAGCAATCAAAAGTTGCCCTAAG‑ACTTTCCAGTCCAACTATGTACGGAACAATGCGAGCCTCGTAGCGGAGGCCGCTTCCCGTGGTCACATCTCGTGCCTGACTACTAGTGGAc                                          >  1:46383‑M2/41‑301 (MQ=255)`  
                                                                                                                                                                                                                                                                       `gAAGGAAATGATTGTCCAGTTCCTGACACACGGTATGGAAGGATTGATGACATTCGTAGTACGTACATCATTTCGTGAGGCCATTAAGGACATGCACGAAGAGTATGCAGATAAGGACTCTTTCAAACAATCTCCTGCAACAGTACGGGAGGTGTTCTGATGTCTGACTACTTGAAGGTCCTGCAAGCAATC‑AAAGTTTCCCTTAGAACTTTCCAGTCAAACTATGTCGGGAACAATCCAGGCTCCTTAGCGAGGCCCGTTTCCCGTGTTCACACCTCGTGCCTGCTTACTGGTGACCGTa                                       >  1:9289/1‑301 (MQ=255)`  
                                                                                                                                                                                                                                                                         `atgctgactcagagtaaagaggacttggttgtatgGAAGGATTGATGACATTCGTAGTACGTACATCATTTCGTGAGGCCATTAAGGACATGCACGAAGAGTATGCAGATAAGGACTCTTTCAAACAATCTCCTGCAACAGTACGGGAGGTGTTCTGATGTCTGACTACCTGAAAGTGCTGCAAGCAATCAAAAGTTGCCCTAAG‑ACTTTCCAGTCCAACTATGTACGGAACAATGCGAGCCTCGTAGCGGAGGCCGCTTCCCGTGGTCACATCTCGTGCCTGACTCCTAGTGGACGTAAc                                     >  1:404051‑M2/36‑301 (MQ=255)`  
                                                                                                                                                                                                                                                                           `tcggagatgtgtataagagacagacacGGTATGGAAGGATTGATGACATTCGTAGTACGTACATCATTTCGTGAGGCCATTAATGACATGCACGAAGAGTATGCAGATAAGGACTCTTTCAAACAATCTCCTGCAACAGTACGGGAGGTGTTCTGATGTCTGACTACCTGAAAGTGCTGCAAGCAATCAAAAGTTGCCCTAAG‑ACTTTCCAGTCCAACTATGTACGGAACAATGCGAGCCTCGTAGCGGAGGCCGCTTCCCGTGGTCACATCTCGTGCCTGACTACTAGTGGACGTAACgg                                   <  1:230441/278‑1 (MQ=255)`  
                                                                                                                                                                                                                                                                           `gAAATTATTGTCCAGTTCCTGACACACGGTATGGAAGGATTGATGACATTCGTAGTACGTACATCATTTCGTGAGGCCATTAAGGACATGCACGAAGAGTATGCAGATAAGGACTCTTTCAAACAATCTCCTGCAACAGTACGGGAGGTGTTCTGATGTCTGACTACCTGAAAGTGCTGCAAGCAATCAAAAGTTGCCCTAAG‑ACTTTCCAGTCCAACTATGTACGGAACAATGCGAGCCTCGTAGCGGAGGCCGCTTCCCGTGGTCACATCTCGTGCCTGACTACTAGTGGACGTAACgg                                   <  1:154112/301‑1 (MQ=255)`  
                                                                                                                                                                                                                                                                            `ctgactcagagtaaagaggacttggttgtatgGAAGGATTGATGACATTCGTAGTACGTACATCATTTCGTGAGGCCATTAAGGACATGCACGAAGAGTATGCAGATAAGGACTCTTTCAAACAATCTCCTGCAACAGTACGGGAGGTGTTCTGATGTCTGACTACCTGAAAGTGCTGCAAGCAATCAAAAGTTGCCCTAAG‑ACTTTCCAGTCCAACTATGTACGGAACAATGCGAGCCTCGTAGCGGAGGCCGCTTCCCGTGGTCACATCTCGTGCCTGACTACTAGTGGACGTAACGGt                                  <  1:397519‑M2/269‑1 (MQ=255)`  
                                                                                                                                                                                                                                                                                `gATTGTCCAGTTCCTGACACACGGTATGGAAGGATTGATGACATTCGTAGTACGTACATCATTTCGTGAGGCCATTAAGGACATGCACGAAGAGTATGCAGATAAGGACTCTTTCAAACAATCTCCTGCAACAGTACGGGAGGTGTTCTGATGTCTGACTACCTGAAAGTGCTGCAAGCAATCAAAAGTTGCCCTAAG‑ACTTTCCAGTCCAACTATGTACGGAACAATGCGAGCCTCGTAGCGGAGGCCGCTTCCCGTGGTCACATCTCGTTCCTGACTACTATTGGACGTAACGGTGgcg                              >  1:200899/1‑301 (MQ=255)`  
                                                                                                                                                                                                                                                                                    `gTCCAGTTCCTGACACACGGTATGTAAGGATTGCTGACATTCGTAGTACGTACATCATTTCGTGCGGCCATTAAGGACATGCACGAAGAGTATGCAGATAAGGACTCTTTCAAACAATCTCCTGCAACAGTACGGGAGGTGTTCTGATGTCTGACTACCTGACAGTGCTGCAAGCAATCAAACGTTGCCCTAAG‑ACTTTCCAGTCTAACTATGTACGGAACAATGCGAGCCTCGTAGCGTAGGCCGCTTCCCGTGGTCACATCTCGTGCCTGACTACTCGTTGACGTAATGTTGGCGCTTg                          >  1:372832/1‑301 (MQ=255)`  
                                                                                                                                                                                                                                                                                             `cTGACACACGGTATGGAAGGATTGATGACATTCGTAGTACGTACATCATTTCGTGAGGCCATTAAGGACATGCACGAAGAGTATGCAGATAAGGACTCTTTCAAACAATCTCCTGCAACAGTACGGGAGGTGTTCTGATGTCTGACTACCTGAAAGTGCTGCAAGCAATCAAAAGTTGCCCTAAG‑ACTTTCCAGTCCAACTATGTACGGAACAATGCGAGCCTCGTAGCGGAGGCCGCTTCCCGTGGTCACATCTCGTGCCTGACTACTAGTGGACGTAGCGGTGGCGCTTGGGAAATCAc                 <  1:222646/301‑1 (MQ=255)`  
                                                                                                                                                                                                                                                                                             `cTGACACACGGTATGGAAGGATTGATGACATTCGTAGTACGTACATCATTTCGTGAGGCCATTAAGGACATGCACGAAGAGTATGCAGATAAGGACTCTTTCAAACAATCTCCTGCAACAGTACGGGAGGTGTTCTGATGTCTGACTACCTGAAAGTGCTGCAAGCAATCAAAAGTTGCCCTAAA‑ACTTTCCAGTCCAACTATGTACGGAACAATGCGAGCCTCGTAGCGGAGGCCGCTTCCCGTGGTCACATCTCGTGCCTGACTACTAGTGGACGTAACGGTGGCGCTTGGGAAATCAc                 >  1:139506/1‑301 (MQ=255)`  
                                                                                                                                                                                                                                                                                                       `gtatgGAAGGATTGATGACATTCGTAGTACGTACATCATTTCGTGAGGCCATTAAGGACATGCACGAAGAGTATGCAGATAAGGACTCTTTCAAACAATCTCCTGCAACAGTACGGGAGGTGTTCTGATGTCTGACTACCTGAAAGTGCTGCAAGCAATCAAAAGTTGCCCTAAG‑ACTTTCCAGTCCAACTATGTACGGAACAATTCGAGCCTCGTAGCGGAGGCCGCTTCCCGTGGTCACATCTCGTGCCTGACTACTAGTGGACGTAACGGTGGCGCTTGGGAAATCACTGCTTCCGGt       >  1:630855‑M2/6‑301 (MQ=255)`  
                                                                                                                                                                                                                                                                                                       `gtatgGAAGGATTGATGACATTCGTAGTACGTACATCATTTCGTGAGGCCATTAAGGACATGCACGAAGAGTATGCAGATAAGGACTCTTTCAAACAATCTCCTGCAACAGTACGGGAGGTGTTCTGATGTCTGACTACCTGAAAGTGCTGCAAGCAATCAAAAGTTGCCCTAAG‑ACTTTCCAGTCCAACTATGTACGGAACAATGCGAGCCTCGTAGCGGAGGCCGCTTCCCGTGGTCACATCTCGTGCCTGACTACTAGTGGACGTAACGGTGGCGCTTGGGAAATCACTGCTTccgtt       >  1:540660‑M2/6‑299 (MQ=255)`  
                                                                                                                                                                                                                                                                                                       `gtatgGAAGGATTGATGACATTCGTAGTACGTACATCATTTCGTGAGGCCATTAAGGACATGCACGAAGAGTATGCAGATAAGGACTCTTTCAAACAATCTCCTGCAACAGTACGGGAGGTGTTCTGATGTCTGACTACCTGAAAGTGCTGCAAGCAATCAAAAGTTGCCCTAAG‑ACTTTCCAGTCCAACTATGTACGGAACAATGCGAGCCTCGTAGCGGAGGCCGCTTCCCGTGGTCACATCTCGTGCCTGACTACTAGTGGACGTAACGGTGGCGCTTGGGAAATCACTGCTTCcggc       >  1:471907‑M2/6‑300 (MQ=255)`  
                                                                                                                                                                                                                                                                                                          `tgGAAGGATTGATGACATTCGTAGTACGTACATCATTTCGTGAGGCCATTAAGGACATGCACGAAGAGTATGCAGCTAAGGACTCTTTCAAACAATCTCCTGCAACAGTACGGGAGGTGTTCTGATGTCTGACTACCTGAATGTGCTGCAAGCAATCAAAAGTTGCCCTAAG‑ACTTTCCAGTCCACCTATGTACGGAACAATGCGAGCCTCTTAGCGGTGGCTGCTTCCCGTGGTCACATCTCGTGCCTTACTACTAGTGGACGTAACGGTGGCGCTTGGGACATCACTGCTTCCGGTACt    >  1:178728‑M2/3‑301 (MQ=255)`  
                                                                                                                                                                                                                                                                                                            `gAAGGATTGATGACATTCGTAGTACGTACATCATTTCGTGAGGCCATTAAGGACATGCACGAAGAGTATGCAGATAAGGACTCTTTCAAACAATCTCCTGCAACAGTACGGGAGGTGTTCTGATGTCTGACTACCTGAAAGTGCTGCAAGCAATCAAAAGTTGCCCTAAG‑ACTTTCCAGTCCAACTATGTACGGAACAATGCGAGCCTCGTAGCGGAGGCCGCTTCCCGTGGTCACATCTCGTGCCTGACTACTAGTGGACGTAACGGTGGCGCTTGGGAAATCACTGCTTCCGGTACTCg  >  1:736122/1‑301 (MQ=255)`  

                                                                                                                                                                                                                                                                                                            `|`                                                                                                                                                                                                                                                                                                               
`TGACATTCTTGCGGTTGTGGTTGACGAAGATGTGCTTATTTTCACATGGGACGAAGTAAAAGGTAAGACATCCATGTGTGTCGGCAAGAGAAACAAAGGCATAAAACTATAGGAGAAATTATTATGGCTATGACAAAGAAATTTAAAGTGTCCTTCGACGTTACCGCAAAGATGTCGTCTGACGTTCAGGCAATCTTAGAGAAAGATATGCTGCATCTATGTAAGCAGGTCGGCTCAGGTGCGATTGTCCCCAATGGTAAACAGAAGGAAATGATTGTCCAGTTCCTGACACACGGTATGGAAGGATTGATGACATTCGTAGTACGTACATCATTTCGTGAGGCCATTAAGGACATGCACGAAGAGTATGCAGATAAGGACTCTTTCAAACAATCTCCTGCAACAGTACGGGAGGTGTTCTGATGTCTGACTACCTGAAAGTGCTGCAAGCAATCAAAAGTTGCCCTAAG‑ACTTTCCAGTCCAACTATGTACGGAACAATGCGAGCCTCGTAGCGGAGGCCGCTTCCCGTGGTCACATCTCGTGCCTGACTACTAGTGGACGTAACGGTGGCGCTTGGGAAATCACTGCTTCCGGTACTCG  >  NC_001604.1/16728‑17328`

  

Alignment Legend

Aligned base mismatch/match (shaded by quality score): `ATCG/ATCG` `< 3 ≤` `ATCG/ATCG` `< 14 ≤` `ATCG/ATCG` `< 27 ≤` `ATCG/ATCG` `< 37 ≤` `ATCG/ATCG` `< 38 ≤` `ATCG/ATCG`

Unaligned base: `atcg`    Masked matching base: `atcg`    Alignment gap: `‑`    Deleted base: `‑`

Reads not counted as support for junction

read\_name Not counted due to insufficient overlap past the breakpoint.

read\_name Not counted due to not crossing MOB target site duplication.
